# Tuberculosis in Children

**DOI:** 10.4084/MJHID.2013.064

**Published:** 2013-11-04

**Authors:** Susanna Esposito, Claudia Tagliabue, Samantha Bosis

**Affiliations:** 1 Pediatric Highly Intensive Care Unit, Department of Pathophysiology and Transplantation, Università degli Studi di Milano, Fondazione IRCCS Ca’ Granda Ospedale Maggiore Policlinico, Milan, Italy.

## Abstract

Tuberculosis (TB) in children is a neglected aspect of the TB epidemic despite it constituting 20% or more of all TB cases in many countries with high TB incidence. Childhood TB is a direct consequence of adult TB but remains overshadowed by adult TB because it is usually smear-negative. Infants and young children are more likely to develop life-threatening forms of TB than older children and adults due to their immature immune systems. Therefore, prompt diagnoses are extremely important although difficult since clinical and radiological signs of TB can be non-specific and variable in children. Despite undeniable advances in identifying definite, probable, or possible TB markers, pediatricians still face many problems when diagnosing TB diagnosis. Moreover, curing TB can be difficult when treatment is delayed and when multi-drug resistant (MDR) pathogens are the cause of the disease. In these cases, the prognosis in children is particularly poor because MDR-TB treatment and treatment duration remain unclear. New studies of diagnostic tests and optimal treatment in children are urgently needed with the final goal of developing an effective anti-TB vaccine.

## Introduction

The impact of tuberculosis (TB) worldwide remains a serious concern with an estimated 8.7 million new cases (13% co-infected with HIV) and 1.4 million deaths due to TB (430,000 in HIV-infected individuals) in 2011.^1^ Assessing the impact of TB in children (<15 years of age) is particularly challenging since there is no universal diagnostic algorithm. The identification of TB cases in children usually results from a combination of clinical criteria and a non-specific TB test. In 2011, an estimated 490,000 TB cases occurred among children (about 6% of the all cases). Each year, 64,000 children die from TB, making it one of the top ten causes of childhood death.^2^

The global burden of childhood TB is under-reported due to paucibacillary disease which makes diagnosis by sputum smear microscopy and culture difficult.^1^,^3^ In 2007, the World Health Organization (WHO) showed that smear-positive TB in children (<14 years of age) accounted for 0.6–3.6% of reported cases.^1^ These data underestimate the true burden of pediatric TB since incidence is estimated using smear-positive cases. The majority of cases in children less than 12 years of age are smear-negative, and smears are seldom performed in high-burden countries. In low-burden countries, childhood TB constitutes about 5% of TB cases compared to the 20–40% in high-burden countries.

HIV infection has significantly impacted the epidemiology and severity of childhood TB since HIV-infected children have an increased risk of developing and disseminating TB. Initiating highly active antiretroviral therapy (HAART) reduces the risk for TB, but the risk still remains higher than that in HIV-uninfected children.^1^ Moreover, children living in a household with an HIV-infected adult also have a higher risk for TB exposure and infection.^1^

Multidrug-resistant TB (MDR-TB) is another important issue for childhood TB that affects global TB control.^1^,^4^ The global estimate of pediatric MDR-TB is around 40,000 cases per year.^1^,^5^,^6^ Almost 60% of these cases were in India, China, and Russia. The drugs recommended for these cases are “off-label” for children. Thus, MDR-TB is also a problem for children in close contact with adults who have MDR-TB.

The aim of this review is to describe the current knowledge of the clinical presentation, diagnosis, and treatment of TB in children. In doing so, it is also important to discuss future research and development priorities that will help improve the prevention and management of childhood TB.

## Clinical Features of Childhood TB

Infants and young children are more likely than older children and adults to develop life-threatening forms of TB disease (i.e., disseminated TB and TB meningitis), and because of their age, pediatric TB acts as a surrogate for identifying recent transmission.^7^,^8^,^9^ The greatest number of TB cases is seen in children less than 5 years of age and adolescents older than 10 years of age.

*Mycobacterium tuberculosis* is spread through aerosolized particles by subjects with pulmonary TB during expiratory efforts, such as coughing, sneezing, speaking, or singing. Apart from conditions where drainage is present, extrapulmonary TB is not transmitted.^10^ The incubation period, which usually lasts from 2 to 12 weeks, is the time that elapses between infection and identification of a primary lesion or a positive tuberculin skin test (TST). During the first four years of life, concomitant HIV infection or other clinical conditions that impair the immune system represent risk factors for TB progression.^11^ In addition, malnutrition is a predictor of TB and is associated with worse outcomes. This is supported by several lines of evidence, including the role of vitamin D receptor genotypes, and malnutritional effects on immune development.^12^

First, it is important to clarify whether a child has latent TB infection (LTBI) or TB.^13^ Children with LTBI do not show any symptoms of the disease, are not infectious, and cannot spread the bacteria. If the bacteria activate, multiply, and overcome the immune response, children can transition from LTBI to TB. This transition occurs more frequently and faster (within weeks) during childhood.

Children with TB have symptoms that result from the active bacterial multiplication which destroys body tissue in the patient. According to the WHO.^1^ the most common signs and symptoms of childhood TB are: chronic cough, defined as an unremitting cough that has been present and has not improved for more than 21 days with or without wheezing; fever, defined as a body temperature >38°C for 14 days, when other common causes, including pneumonia, have been excluded; objectively documented weight loss or failure to thrive; feelings of sickness or weakness, lethargy, and/or reduced playfulness; and night sweats. Since TB can involve any site within the body, it can produce symptoms and findings that are systemic rather than specifically associated with the organ or tissues involved. Fever is the most easily quantified systemic effect in children.^7^ Other systemic effects include: hematologic manifestations, especially increases in peripheral blood leukocyte count and anemia which are both common in disseminated TB, and electrolyte imbalance, especially hyponatremia which is caused by the production of an antidiuretic hormone-like substance within affected lung tissue.^7^

When TB disease is diagnosed, it must be classified as either pulmonary or extrapulmonary TB. Pulmonary TB originates from an exogenous reinfection or endogenous LTBI reactivation. Pulmonary parenchymal disease and intrathoracic adenopathy are the most common clinical manifestations of pediatric TB and account for 60–80% of all cases.^10^ The majority of children who acquire a M. tuberculosis infection have pulmonary infection weeks after acquisition with no signs, symptoms, or radiographic abnormalities.^8^ Inflammation is initially localized with a nonspecific infiltrate that can be identified radiographically. Within days, the infection spreads to regional lymph nodes, causing them to disproportionately swell compared to the parenchymal focus. Newly-infected older children are asymptomatic in 80–90% of cases, while newly-infected infants experience symptoms or have radiographic findings of pulmonary TB in 40–50% of cases.^8^

Symptoms are usually mild, including low-grade fever and cough. The cough is usually unproductive, and mild dyspnea is common in infants. Focal wheezing and respiratory distress, especially in infants, can also manifest. The signs needed to confirm the diagnosis via chest radiograph and computed tomography (CT) are typically absent. Hilar and mediastinal adenopathy is predominant although not always discernible by plain radiographs.^14^ Any lobe of the lung can be involved, and in 25% of the cases, the TB is multilobar. In primary TB (i.e., as the result of a recent infection), infection is generally seen in infiltrate from the middle or lower and is often associated with ipsilateral hilar adenopathy.^8^,^14^ Additionally, the progressive lymph node enlargement may cause atelectasis due to compression.

Endogenous TB that develops from LTBI reactivation usually causes abnormalities in the upper lobes of one or both lungs.^8^,^14^ As the localized infection progresses, cavitation and collapse or consolidation may occur.^8^,^14^ A local pleural reaction or effusion is common, and obstructive signs and symptoms may be evident if an endobronchial lesion develops. Pulmonary radiographic findings range from normal to diverse abnormalities such as: hilar, subcarinal, or mediastinal lymphadenopathy; atelectasis or infiltrate in the lobe; pleural effusion or cavitary lesions; or miliary disease.^13^,^16^,^17^

Extrapulmonary TB is typically harder to diagnose than pulmonary TB because clinicians are less familiar with this form. Extrapulmonary TB involves relatively inaccessible sites; therefore, fewer bacilli can cause greater damage, and invasive procedures are frequently required for diagnosis. Although uncommon, some symptoms are highly suggestive of extrapulmonary TB and require further investigation: gibbus (especially recent onset) from vertebral TB, enlarged and painless joints or cervical lymphadenopathy with fistula formation, meningitis or pleural effusion that does not respond to antibiotics, pericardial effusion, a distended abdomen with ascites, and signs of tuberculin hypersensitivity (e.g., phlyctenular conjunctivitis or erythema nodosum).^8^,^10^ TB lymphadenitis is also extremely common in children. Lymph nodes, usually supraclavicular or cervical, are swollen, painless, and firm.^7^,^8^ The involvement of neck nodes is secondary to disease spread from a pulmonary focus.^7^,^8^

Central nervous system involvement is quite common in children and is secondary to lympho-haematogenous spread. The most common manifestation in children is meningitis, primarily at the base of the brain.^15^,^16^ Clinical presentation can range from a non-specific headache with no focal signs to meningitis, neck stiffness, and focal signs, or even coma, hemiplegia, and signs of raised intracranial pressure. Disease outcome depends on symptom severity. Lumbar punctures are used to diagnose this type of TB, and typical findings include low sugar concentrations, high protein levels, and increased lymphocyte counts. TB stains are positive in only a minority of cases.^15^–^17^ Head CT scans should be performed before lumbar punctures if physical examinations find a focus or if increased intracranial pressure is suspected.^15^–^17^

Miliary or disseminated TB occurs because host defenses are unable to contain the TB infection. In children, protean clinical manifestations occur, and children can present with high fever, cachexia, respiratory distress, nodular changes in chest X-rays, and hepato-splenomegaly.^18^ This type of TB is most common in children after recently acquiring TB because their defenses cannot contain the bacilli.

Pleural TB manifests primarily as empyema as a consequence of a large number of organisms spilling into the pleural space. This is usually due to the rupturing of a cavity or adjacent parenchymal focus via a broncopleural fistula.^17^ Pericardial TB presents as either normal TB infection or as pericardial inflammation that causes pain, effusion, and eventually hemodynamic effects.^17^

Approximately 1% of young children with TB present with pain from a bony focus.^17^ The symptoms are subtle, and diagnostic evaluations are often not undertaken until the process is advanced. Vertebral disease leads to bone destruction and collapse resulting in spinal malformation if diagnosis is delayed.^17^

Abdominal TB can involve any intrabdominal organ as well as the peritoneum. The areas involved determine its clinical manifestations. Children usually present with fever, anorexia, weight loss, lymphadenopathy, and ascites.^19^

The clinical symptoms of children with MDR-TB cannot be differentiated from those of children with drug-susceptible TB.^4^

## Diagnosis

Diagnosing TB in children is difficult because of non-specific and variable clinical and radiological signs, especially in patients younger than 4 years and those with HIV infection.^20^

Diagnosing and treating LTBI is important for reducing the risk of progressing to active TB. The current definition of LTBI includes a diverse range of individuals, from those who have completely cleared the infection to those who are incubating actively replicating bacteria in the absence of clinical symptoms.^21^ Thus, the precise definition of LTBI presently remains an open question among the scientific community.^22^ This explains why there is no gold standard for LTBI diagnosis tests. Before 2001, the tuberculin skin test (TST) was the only commercially-available immunologic test for identifying *M. tuberculosis* infection.^23^ It is used worldwide for diagnosing both LTBI and active TB, but it has some limitations. TST must be administered properly by the Mantoux method consisting of an intradermal injection of 0.1 mL tuberculin-purified protein derivative into the forearm’s volar surface.^23^Table 1 summarizes the three cut-offs that define a positive TST. Although positive results are usually associated with an increased risk for current or future active TB, TST can also give false positives from non-tuberculous mycobacteria or in subjects vaccinated with Bacille Calmette-Guerin (BCG). This is the result of the TST also testing for antigens that are found in non-tuberculous mycobacteria and the vaccine.^20^

Due to these limitations, interferon gamma (IFN-γ) release assays (IGRAs) were developed to also detect *M. tuberculosis*.^20^,^23^ These new tests assess the release of IFN-γ in response to synthetic overlapping peptides representing *M. tuberculosis* proteins (i.e., ESAT-6 and CFP-10). These peptides are absent in the BCG vaccine strains and most non-tuberculous mycobacteria. The QuantiFERON-TB Gold In Tube (QFT-GIT) assay uses positive and negative controls and test antigen, which consists of a mixture of 14 peptides that represent the entire amino acid sequences of ESAT-6, CFP-10, and TB7.7. Fresh blood from the patient is collected, mixed separately with these reagents, and then incubated for 16–24 hours.^24^,^25^ The negative-control tube contains heparin alone while the positive contains heparin, dextrose, and phytohemaglutinin. The plasma IFN-γ concentration is determined using by enzyme-linked immunosorbent assay (ELISA), and the TB response is calculated as the difference between the IFN-γ concentration in plasma stimulated by the antigens and that in plasma incubated without antigens.^24^,^25^

A new IGRA, the T-Spot, was approved in 2008. In this test, peripheral blood mononuclear cells are incubated with a mixture of two peptides (ESAT-6 and CFP-10) that stimulate IFN-γ production and control substances.^24^,^25^ An enzyme-linked immunospot assay (ELISpot) is then used to detect the increase in the number IFN-γ-secreting cells (represented as a spot in each test well).^25^

The use of IGRAs in children is limited, and studies evaluating the performance of these tests in pediatric populations are scant. Bua et al. evaluated QFT-GIT performance compared to TST for diagnosing TB in 105 Italian immunocompetent children with a history of contact with patients who had active pulmonary TB.^23^ All children underwent a TST and QFT-GIT, and chest X-rays and microbiological examinations were performed in only those with positive results. QFT-GIT-positive/TST-positive and QFT-GIT-negative/TST-negative were found in 18% and 72%, respectively, for an overall concordance of 90% between the two tests. This indicated that both tests could be used to identify infected children. Moyo et al. also compared TST and QFT-GIT results from 400 children (<3 years of age) with suspected TB and demonstrated a similarly high concordance (94%).^24^ QFT-GIT and TST sensitivity was 38% and 35%, respectively, and the specificity was 81% and 84%, respectively, suggesting that QFT-GIT and TST were equivalent for diagnosing TB in young children from high-burden settings.^24^ IGRAs have been designed to be more specific for LTBI diagnoses than for TST in patients who have been exposed to non-tuberculous mycobacteria or vaccinated. Therefore, based on these studies, the performance of TB IGRAs have not been sufficiently demonstrated in children and are not sufficient to exclude or confirm active TB diagnoses.^25^ Improved TB diagnostic methods are needed to reduce TB transmission and mortality in children.

The microbiological isolation of *M. tuberculosis* is very important for identifying active TB and also drug-resistant TB. In children, a microbiological diagnosis is made in only 20–40% of cases due to the disease’s paucibacillary nature.^26^ Collecting specimens from children is also very difficult, especially from the youngest patients who cannot produce adequate sputum. Sequential gastric lavage (GL) obtained over two or three consecutive days after the child awakens and before eating or drinking is recommended for diagnosing pediatric TB.^20^,^26^ Jiménez et al. performed a prospective pilot study in 22 children with suspected pulmonary TB hospitalized in Madrid to assess the safety and diagnostic yield of induced sputum (IS) with GL collected on three consecutive days. All samples were tested by *M. tuberculosis* staining, culture, and polymerase chain reaction. *M. tuberculosis* was identified from GL and IS in 47.1% and 41.2% of samples, respectively. This indicates that IS is a safe and well-tolerated exam for infants.^26^

## Therapy

The main goals of successful TB treatment are curing the patient and limiting M. tuberculosis transmission in the community. The current recommended treatment regimens for adults and children are essentially the same. They are based on a combination of drugs that eliminate the mycobacteria via different mechanisms to prevent the emergence of resistant organisms while having minimal toxicity.^27^,^28^ Isoniazid (INH) and rifampicin (RIF) are the most potent first-line bactericidal drugs. They kill the bacteria and rapidly decrease microbial loads, leading to clinical improvements as well as hindering disease progression and transmission.^27^,^28^ Additionally, RIF and pyrazinamide (PZA) are sterilizing drugs that eradicate slow-replicating organisms. The combination of bactericidal with sterilizing drugs and the addition of a fourth drug, such as ethambutol (EMB) or streptomycin (S), protects against the emergence of drug-resistant *M. tuberculosis*.^27^,^28^ Because of the high risk of disseminated TB in infants and children younger than 4 years, TB treatment should be started as soon as a TB diagnosis is suspected.^27^,^28^ For LTBI, INH administered daily for 6–9 months or RIF for 4 months, for those who cannot receive INH, is recommended as prophylaxis.^27^–^29^

Basic drug regimens that include INH, RIF, PZA, and EMB are recommended for treating children with pulmonary tuberculosis caused by organisms known or presumed to be susceptible to those drugs.^29^ EMB is not used routinely in children less than 8 years of age because it can cause optic neuritis, which can lead to irreversible blindness if EMB is not discontinued when visual problems occur. However, since the toxicity is related to the dosage and duration of therapy, EMB can be safely used at the recommended dosage for the initial two months of treatment, even in children too young for routine eye examinations.^27^–^29^ According to recommendations, each regimen has an initial phase of two months with 3 or 4 drugs, followed by a continuation phase of either 4 or 7 months with INH and RIF.

In general, extrapulmonary TB in children can be treated with the same regimen as pulmonary disease. The exceptions are disseminated TB and tuberculous meningitis. In these cases, 9–12 months of therapy are recommended, and steroids in the first weeks of treatment also appear to be useful.^29^ The recommended first-line drug dosages for children are listed in Table 2.

Since anti-TB drug production has focused on adults, most drugs are in the form of tablets which have some advantages in poor settings because they can be more easily transported and stored than liquid drugs. However, infants and young children cannot swallow the tablets, and in these patients, galenic formulations must be prepared from the tablets.^30^,^31^ Hepatotoxicity is the major drug-related adverse event to these drugs and is frequently associated with INH and RIF.^29^ In these cases, one of the two drugs should be interrupted. However, serious adverse events due to TB-drugs are rare in children.^30^ In situations where several first-line agents cannot be used because of intolerance, regimens based on principles described for treating MDR-TB should be used.^32^

The optimal treatment for pulmonary TB in children with HIV infection is unknown. The major authorities in the United States recommend at least three drugs for initial therapy and a therapy duration of at least 9 months. No data support this recommendation, however.^29^

The emergence of drug resistance is a major public health concern since it is associated with treatment failure and increased mortality.^30^,^31^Table 3 summarizes second line drugs for TB treatment. Generally, children affected by TB are treated for drug-susceptible TB; however, once treatment begins failing, microbiological data are positive for MDR-TB, or a MDR-TB source case is identified, they must be treated with second-line drugs. MDR-TB therapy in children is complex because data regarding drug regimen, dosage, safety, and therapy duration are scant and extrapolated from adult data.^30^,^31^ In a retrospective study conducted in South Africa, 111 children with MDR-TB (median age of 50 months) were examined. Of these children, 91 (82%) had a favorable treatment outcome (67% with MDR-TB regimen and 3.3% with first-line drugs) with a mortality of 12%.^32^ Treatment regimens lasted about 18 months with a median of 7 drugs, including one injectable drug, for about 6 months. The authors demonstrated that children with confirmed MDR-TB tended to be HIV-positive (43 of 100 children [43%] tested; 27 of 43 [64%] with severe immunosuppression) and have severe chest radiographic features at diagnosis [32]. Moreover, a multivariate analysis showed that malnutrition, HIV infection, and extrapulmonary disease predicted mortality in children with MDR-TB.^32^ In Italy, Mauro et al. recently described a case of MDR-TB in a 3-year-old child with tuberculous meningitis who had strains resistant to INH, RIF, and S.^33^ The authors used an alternative regimen of ciprofloxacin, PZA, and ethionamide with good results.

Levofloxacin, moxifloxacin, or gatifloxacin may be useful in alternative regimens, but the potential role of a fluoroquinolone and the optimal treatment length have not been defined in children.^29^,^32^ A recent study enrolled 9 patients (aged 6 month to 13 years) with pulmonary TB.^34^ All patients were treated with moxifloxacin as part of their regimen even though one child did not show evidence of TB resistance. The authors demonstrated that all patients were cured (one was lost at follow up) and adverse events (arthritis) in two patients. This suggests that moxifloxacin should be considered for treating TB, especially MDR-TB, in children.^34^ Gegia et al. evaluated the characteristics and treatment outcomes of pediatric MDR-TB in 45 children (aged <16 years) with confirmed or suspected MDR-TB.^35^ The second-line drugs used in this study included PZA, capreomycin (CPM), levofloxacin, prothionamide, cycloserine, and p-aminosalicylic acid (PAS). The authors demonstrated that 77.1% of patients were successfully treated.^35^

A recent systematic review and meta-analysis of eight studies reporting the treatment outcomes for children with MDR-TB from Peru, Spain, the United States, and South Africa showed that the overall treatment success with second-line drugs was 81.7%.^36^ Response to treatment was higher for studies that included injectable drugs in their regimen. Overall, 5.9% of patients died and 39% had adverse events.^36^ These events occurred as nausea and vomiting in most cases and as severe events, such as hearing loss (from amikacin and CPM), psychiatric effects (from cycloserine), and hypothyroidism (from ethionamide), in other cases.^36^

The WHO considers linezolid an alternative drug for MDR-TB treatment. It has been successfully used in adults, but data in children are scant. Rose et al. conducted a retrospective study in seven children (aged 1–13 years) with MDR-TB who were treated with drug regimens that included linezolid.^37^ Three of these patients were HIV-infected. Non-HIV-infected patients did not experience adverse events, whereas all HIV-positive patients had adverse reactions, one of which was life-threatening.^37^ Adverse events included pancreatitis, peripheral neuropathy, anemia, and leucopenia. Four children were cured, and the others were still receiving therapy but improving. This suggests that regimens containing linezolid should also be considered for children with MDR-TB as long as the adverse events and high monetary costs are taken into account.^36^

Together, these data suggest that treating pediatric TB should be done by or in close consultation with a pediatric infectious diseases specialist. This is particularly true for cases of drug-resistant TB because second-line regimens often are the patient’s last hope for being cured.

## Conclusions

Despite undeniable advances in identifying markers of definite, probable, or possible TB in recent years, many problems pediatricians face in managing TB remain unsolved. The most important difficulty lies in early diagnosis because treatment can completely cure the majority of cases where TB is suspected early. This is only with the assumption that the pathogens are fully drug susceptible and that patient compliance in treatment is ideal. Achieving a cure is more difficult when treatment is delayed and when MDR pathogens are the cause of the disease. In these cases, prognosis is poor, particularly in children, because what can be done to treat MDR-TB is unclear. New studies of diagnostic tests and optimal treatment for children are urgently needed with the final goal of developing an effective anti-TB vaccine. In the meantime, an aggressive attitude must be adopted for both diagnosing and treating a child with suspected TB because TB can be a devastating disease for children.

## Figures and Tables

**Table 1:** The three cut-offs of positive TST results in children

Diameter of induration	TB-positive patients
≥5 mm	Children in close contact with a known or suspected infectious case of TB Children with suspected TB (i.e., chest radiographs consistent with active or previously active TB or clinical evidence of TB) Children who are immunosuppressed (i.e., receiving immunosuppressive therapy or with immunosuppressive conditions)
≥10 mm	Children at increased risk of disseminated disease (i.e., those <4 yrs old or those with concomitant medical conditions, including Hodgkin’s disease, lymphoma, diabetes mellitus, chronic renal failure, or malnutrition) Children with increased risk of TB (i.e., those born in a country with a high TB prevalence, those who travel to a country with a high TB prevalence, those with parents born in a country with a high TB prevalence, or those frequently exposed to adults with risk factors for TB)
≥10 mm	Children ≥4 years old with no known risk factors

**Table 2:** Recommended first-line TB drugs for infants and children

Drug	Daily dosage (max)
Isoniazid	10–15 mg/Kg (300 mg)
Rifampin	10–20 mg/Kg (600 mg)
Rifabutin	Appropriate dosage unknown
Pyrazinamide	15–30 mg/Kg (2.0 g)
Ethambutol	15–20 mg/Kg (1.0 g)

Adapted from the American Thoracic Society, CDC, and Infectious Diseases Society of America.^29^

**Table 3:** Recommended daily dosages of second-line TB drugs for infants and children

Drug	Dosage
Ethionamide	20 mg/Kg/24 h (max 1.0 g/day) orally as a single daily dose
Cycloserine	10–15 mg/kg/24 h (max 1.0 g/day) orally as a single daily dose
Steptomycin	20–40 mg/kg/24 h (max 1.0 g/day) i.m. or i.v. as a single daily dose
Para-amino-salicylic acid	200–300 mg/Kg/24 h orally in 2–4 doses
Capreomycin	15–30 mg/kg/24 h (max 1000 mg) orally as a single daily dose
Amikacin and	15–30 mg/kg/24 h (max 1.0 g/day)
Kanamycin	i.m. or i.v. as a single daily dose
Ofloxacin	15–20 mg/kg/24 h (max 800 mg) orally as a single daily dose
Levofloxacin	7.5–10 mg/kg/24 h (max 500 mg) orally as a single daily dose
Moxifloxacin	7.5–10 mg/kg/24 h (max 500 mg) orally as a single daily dose
Ciprofloxacin	20–30 mg/kg/24 h (max 1.5 g) orally as a single daily dose
